# The arrhythmogenic cardiomyopathy-specific coding and non-coding transcriptome in human cardiac stromal cells

**DOI:** 10.1186/s12864-018-4876-6

**Published:** 2018-06-25

**Authors:** Johannes Rainer, Viviana Meraviglia, Hagen Blankenburg, Chiara Piubelli, Peter P. Pramstaller, Adolfo Paolin, Elisa Cogliati, Giulio Pompilio, Elena Sommariva, Francisco S. Domingues, Alessandra Rossini

**Affiliations:** 1 Institute for Biomedicine, Eurac Research, Affiliated Institute of the University of Lübeck, Viale Druso 1, 39100 Bolzano, Italy; 2 Treviso Tissue Bank Foundation, Piazzalo Ospedale 1, 31100 Treviso, Italy; 3 Vascular Biology and Regenerative Medicine Unit, Centro Cardiologico Monzino IRCCS, via Parea 4, 20138 Milan, Italy

**Keywords:** Arrhythmogenic cardiomyopathy, Cardiac stromal cells, microRNA expression, Gene expression, Genomics, Transcriptome profiling

## Abstract

**Background:**

Arrhythmogenic cardiomyopathy (ACM) is a genetic autosomal disease characterized by abnormal cell-cell adhesion, cardiomyocyte death, progressive fibro-adipose replacement of the myocardium, arrhythmias and sudden death. Several different cell types contribute to the pathogenesis of ACM, including, as recently described, cardiac stromal cells (CStCs). In the present study, we aim to identify ACM-specific expression profiles of human CStCs derived from endomyocardial biopsies of ACM patients and healthy individuals employing TaqMan Low Density Arrays for miRNA expression profiling, and high throughput sequencing for gene expression quantification.

**Results:**

We identified 3 miRNAs and 272 genes as significantly differentially expressed at a 5% false discovery rate. Both the differentially expressed genes as well as the target genes of the ACM-specific miRNAs were found to be enriched in cell adhesion-related biological processes. Functional similarity and protein interaction-based network analyses performed on the identified deregulated genes, miRNA targets and known ACM-causative genes revealed clusters of highly related genes involved in cell adhesion, extracellular matrix organization, lipid transport and ephrin receptor signaling.

**Conclusions:**

We determined for the first time the coding and non-coding transcriptome characteristic of ACM cardiac stromal cells, finding evidence for a potential contribution of miRNAs, specifically miR-29b-3p, to ACM pathogenesis or phenotype maintenance.

**Electronic supplementary material:**

The online version of this article (10.1186/s12864-018-4876-6) contains supplementary material, which is available to authorized users.

## Background

Arrhythmogenic cardiomyopathy (ACM) is a genetic autosomal disease that affects about 1 in 2000–5000 young people, in particular athletes [1, 2]. It is mainly caused by mutations in genes encoding desmosomal proteins even if other genes have been associated with ACM too [3]. ACM is characterized by cardiomyocyte death and progressive accumulation of fibro-adipose tissue, but the pathogenic mechanisms remain still largely unknown. Abnormal cell-cell adhesion and intracellular signaling, caused by the mutations, are thought to alter Wnt/β-catenin [4] and Hippo [5] pathways resulting in myocyte death, fibro-adipogenesis, gap junction and ion channel remodeling. This leads to myocardium wall thinning, progressive heart failure, inefficient current conduction, and life threatening arrhythmias [6]. For a long time, ACM has been considered a cardiomyocyte-specific disease, but this concept has been questioned when other cell types were found to be implicated in ACM pathogenesis. In particular, cardiac stromal cells (CStCs), an abundant cardiac cell population with differentiation potency [7] and a role in cardiac homeostasis and remodeling during pathological conditions [8], have recently been characterized as a source of adipocytes in ACM [8]. Human primary CStCs from ACM patients express desmosomal proteins and show, compared to healthy controls, a greater propensity to undergo adipogenic differentiation in vitro in a *PKP2*- and Wnt pathway-dependent fashion [8]. Importantly, CStCs can contribute to the formation of adipose tissue in the ACM affected heart.

While extensive characterizations have been performed on the genetic basis of this disease, only few studies have been conducted investigating differences at transcriptional levels [9–11]. The present work addresses, for the first time, the characterization of the ACM-specific coding and non-coding transcriptome in CStCs. To this end we compared expression of genes and miRNAs in cells derived from diagnostic biopsies of ACM patients or controls. In order to reveal biological pathways potentially involved in ACM we performed category enrichment and network analyses on the differentially expressed genes and validated target genes of the deregulated miRNAs.

## Methods

### Biological samples and cell culture

Biopsy samples were obtained from ACM patients’ right ventricle during diagnostic procedures in the area adjacent to the electroanatomical scar. Control samples were obtained from the right ventricle of explanted hearts of cadaveric donors (healthy hearts of organ donors). To limit the variability due to regional differences, we selected small portions of the endocardium in the right ventricle free wall of the donors’ hearts, as this matches the most likely source of ventricular biopsies in ACM patients. All enrolled individuals are males aged between 37 and 64 (mean age for ACM patients: 47.6, standard deviation 8.6, mean age for controls: 48.2, standard deviation 7.8). All ACM patients fulfill definite ACM diagnosis as defined by the Task Force 2010 criteria (either two major criteria or one major and two minor or four minor). *PKP2* mutations in ACM patients were determined by Sanger sequencing. Samples, both from ACM patients and control individuals, were processed, as previously described, to obtain CStCs [7] on which the expression profiling was performed. Adherent cells were cultured, propagated and used between passage 3 and 9. As previously described [7], mesenchymal lineage was confirmed and endothelial and hematopoietic origin was excluded through flow cytometry analysis of surface markers. See Table 1 for an overview of samples, clinical data and experiments in which they were included.

**Table 1:** Characteristics of enrolled subjects

ID	Used for	Mutations	Arrhythmias	Dysfunction,structural and tissue alterations	ECG abnormalities	ACM
ACM1	m,t,mv,tv	*PKP2*:1643delG	PVCs; ventricular fibrillation	RV wall hypokinesia; LV and RV dilation; fibro-adipose substitution	Inverted T-waves	(5/1)
ACM2	t,mv,tv	*PKP2*:c.1881delC	n.c.	n.c.	n.c.	(4/1)
ACM3	t,mv,tv	n.a.	n.c.	n.c.	n.c.	(1/2)
ACM4	m,mv,tv	*PKP2*:2013delC	Arrhythmic storm; sustained VT	RV bulging; RV subendocardic trabeculation; fibro-adipose substitution		(3/1)
ACM5	m,mv	n.a.	Arrhythmic storm; sustained VT	RV wall hypokinesia; fibro-adipose substitution	Inverted T-waves	(2/1)
ACM6	mv,tv	n.a.	PVCs	RV dilation and bulging; RV wall hypokinesia		(2/1)
ACM7	tv,mv	*PKP2*:c.548G > A	n.c.	n.c.	n.c.	(3/0)
ACM8	tv,mv	n.a.	n.c.	n.c.	n.c.	(3/0)
CTRL1	m,t,mv,tv	n.a.				
CTRL2	m,t,mv,tv	n.a.				
CTRL3	m,t,mv,tv	n.a.				
CTRL4	tv	n.a.				
CTRL5	tv	n.a.				
CTRL6	mv,tv					
CTRL7	mv,tv					

All are male individuals aged 37 to 64. *Abbreviations*: *n.a*. Not available, *PVC* Premature ventricular contractions, *VT* Ventricular Tachycardia, *m* Whole genome miRNA screening, *t* Whole genome transcriptome analysis, *mv* miRNA validation, *tv* Transcriptome validation. *n.c.*: not shown because consent for publication not signed. Column *ACM*: number of diagnostic criteria based on ACM task force 2010 in the form (major/minor)

### RNA preparation

RNA was TRIZOL-extracted and quantified with a NanoDrop ND-1000 Spectrophotometer. RNA quality was assessed by an Agilent 2100 Bioanalyzer (Small RNA Kit) for miRNA expression and by an Experion electrophoresis system (RNA High Sense Analysis Kit, Bio-Rad) for gene expression profiling. Only high quality RNAs (A260/280 and A260/230 ratios > 1.8 and an RQI > 9.5) were used for subsequent analyses.

### miRNA expression analysis

miRNA expression was measured by TaqMan Low Density Arrays (TLDA, Human MicroRNA Panel A and B Card Sets v3.0, Applied Biosystems) on 480 ng of total RNA from 3 ACM patients and 3 healthy subjects using a 7900TH System (Applied Biosystems) following manufacturer’s instructions. The complete data set consisted of 24 TLDAs: 2 replicates for each sample and two cards, A and B, for each. Primary data analysis was conducted with SDS RQ manager (Applied Biosystems) applying a manual threshold (0.2) and baseline (based on cycles 10–16). Further analysis was performed in R (version 3.4.2). The data was normalized with the global geometric mean scaling method [12]. miRNA assays were annotated to miRBase v20 using the LifeTech online search tool. Replicated measurements per individual were averaged prior to differential expression analysis and all assays that did not yield a valid measurement in at least 2 out of the 3 individuals in one of the two sample groups (because their measurements were flagged by the SDS software as “Undetermined” or “No amplification in well”) were excluded. This reduced the data set from 768 assayed to 208 detected miRNAs. Significance of differential expression was assessed with the moderated t-test [13], *p*-values were adjusted for multiple hypothesis testing with the method from Benjamini and Hochberg controlling the false discovery rate (FDR) [14]. miRNAs with an adjusted p-value p_adj_ < 0.05 were considered significant.

### High throughput sequencing and data analysis

Paired-end mRNA-sequencing libraries were prepared using the Illumina TruSeq RNA Sample Prep Kit v2-Set A starting from 1 μg of total RNA obtained from CStCs of 3 ACM patients and 3 healthy controls, following manufacturer’s instructions. Samples were multiplexed and sequenced on 4 lanes of an Illumina HiSeq2000 resulting in between 53 and 85 million read pairs per sample and a high average base call quality (Q_phred_ score > 35). Subsequent analysis was performed in R (https://www.r-project.org, version 3.4.2, Bioconductor version 3.5). The 100 nt-long raw reads were filtered removing all reads with 7 or more undetermined nucleotides and subsequently trimmed as soon as 3 of 5 consecutive nucleotides in a read’s sequence had a base-call quality Q_phred_ < 20. Finally, all reads shorter than 41 nt were removed. Alignment of the reads was performed against genome GRCh38 (Ensembl 81) using GSNAP [15].

Gene quantification was performed using GenomicAlignments [16] counting the number of reads falling completely within the exons of a gene, not considering multi-mapping reads or reads with a low alignment quality (phred score < 30). Genes with a normalized read count < 10 in both sample groups were removed, reducing the data set from 60,411 human genes defined in Ensembl to 18,076. Differential gene expression analysis was performed using DESeq2 [17] disabling the automatic pre-filtering. Raw *p*-values were adjusted for multiple hypothesis testing using the method from Benjamini and Hochberg [14]. All genes with an adjusted p-value p_adj_ < 0.05 were considered significant.

### Quantitative reverse transcriptase–polymerase chain reaction

One μg of total RNA was reverse transcribed using the SuperScript® VILO™ cDNA Synthesis Kit (Invitrogen), according to manufacturer’s instructions. cDNA was amplified using TaqMan® Universal PCR Master Mix (Applied Biosystems). For evaluation of *JUP* expression, cDNA was amplified by SYBR-GREEN qPCR. miRNA expression was measured with TaqMan assays for the individual miRNAs. Real-time reactions were performed following manufacturer’s instructions on a CFX96™ RT-qPCR Detection System (Bio-Rad, USA). See extended Material and Methods in the Supplement for the list of primers and assays.

RT-qPCR data analysis was performed in R. Two technical replicates per sample were averaged and normalized against house-keeping control *TBP* (for genes) or miR-28-3p (for miRNAs) to yield the ΔC_q_ values [18]. miR-28-3p was selected due to its stable expression across all samples in the whole genome miRNA expression analysis. Where present, measurements in RNA from the same individual but from different experiments/passages were averaged prior to differential expression analysis. Significance of differential expression was assessed using the Student’s t-test.

### Category enrichment analysis

The GOstats package [19] was used for category enrichment analyses, with categories representing either sets of genes annotated to pathways in the Reactome database, or to terms from one of the 3 Gene Ontologies Biological Process, Cellular Component or Molecular Function. Genes considered to be expressed in the present data set were used as background gene set. The *p*-values from the hypergeometric tests were adjusted for multiple hypothesis testing using the Bonferroni method and pathways/GO terms with an adjusted p-value < 0.05 were considered significant.

### miRNA target gene and host gene analysis

Validated miRNA target gene information was taken from the miRTarbase (version 6.1) [20] and the analysis conducted using our “mirtarbase” R-package (https://github.com/jotsetung/mirtarbase). For identification of potential miRNA host genes/primary transcription units [21] we used our “mirhostgenes” R-package (https://github.com/jotsetung/mirhostgenes) with host gene predictions for miRBase v21 and genes defined in Ensembl version 81.

Systematic evaluation of the downregulation of ACM-miRNA target genes was performed as follows: for each miRNA, the average M-value (representing the differential expression of the gene) was calculated across all of its target genes and compared to average M-values of randomly selected, same-sized, sets of miRNA target genes in a permutation test (1000 permutations). The *p*-value represents the fraction of average M-values of the random gene sets that were < = the average M-value of the miRNA’s target genes (one-tailed test). Background genes were selected randomly among validated (and expressed) target genes of expressed miRNAs. We additionally used a Student’s t-test to test the alternative hypothesis of an average M-value < 0.

### Gene network analysis

Gene interaction networks were defined using information from BioPlex [22] (version 4), mentha [23] (human data release 2015.11.27), STRING [24] (version 10, requiring confidence score of at least 0.9), and a functional similarity network [25] based on Gene Ontology (GO) biological process annotations (GO version 2015.09). Only genes that yielded a read count in the present transcriptome data set or that are expressed in the heart tissue [26] were included in the networks. Clusters within each network were identified using ClusterOne [27] with default parameters, requiring at least four genes per clister in the BioPlex/mentha/STRING network and five genes in the functional similarity network. Full network analysis results are presented on https://gemex.eurac.edu/bioinf/acm/. Network visualizations were created using python-igraph [28] and Cytoscape.js [29] and Cytoscape, GO annotations and enrichments (FDR < 5%, backgrounds comprising all genes in the respective networks) were computed using Dintor [30].

## Results

### ACM-specific miRNAs

**Fig. 1:**
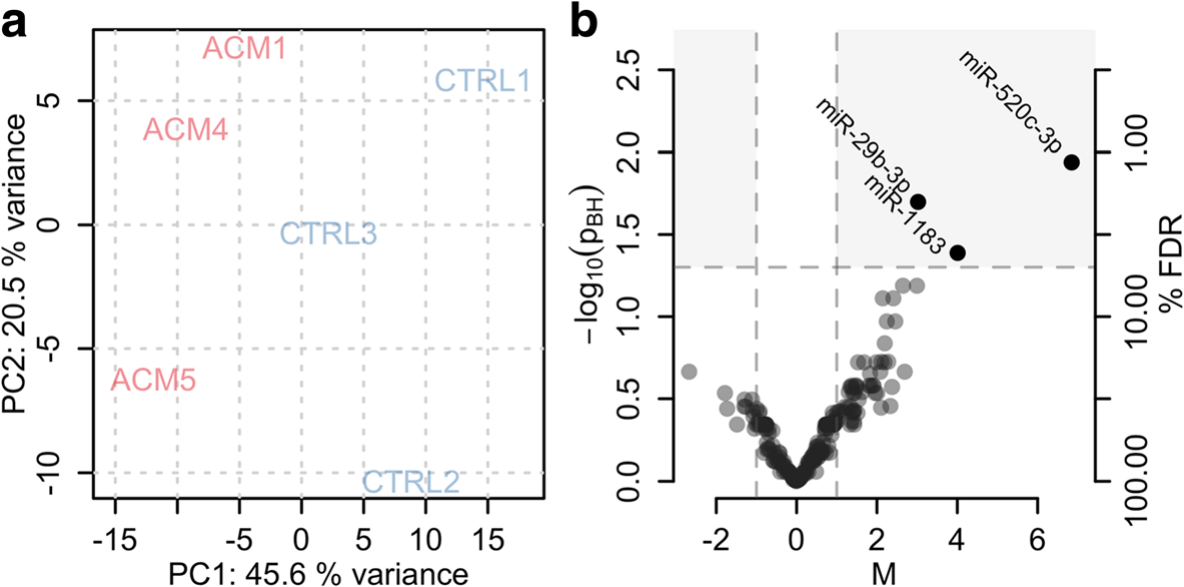
miRNA expression profiling. **a**: Principal component analysis (PCA) result showing the grouping of samples based on their miRNA expression. **b**: Volcano plot showing the extent (M-value, x-axis) and significance (−log_10_ of Benjamini and Hochberg adjusted *p*-values p_adj_, y-axis) of differential expression between ACM and control samples for each miRNA. Positive M-values indicate higher expression in ACM, negative higher expression in control samples

To delineate the miRNA expression profile characteristic for ACM cells, we extracted RNA from CStCs of 3 ACM patients and 3 (sex and age matched) control individuals and subjected it to whole genome miRNA expression profiling. We identified the 3 miRNAs miR-520c-3p, miR-29b-3p and miR-1183 to be significantly differentially expressed at a 5% FDR (i.e. with a p_adj_ < 0.05; Fig. 1b), all of them being more than 4-fold higher expressed in ACM than in control samples (Table 2; the complete results are provided in Additional file 1). For validation, we performed RT-qPCR measurements of miR-520c-3p and miR-29b-3p in RNA samples from the same 3 ACM and 3 control individuals, but from different passages in two separate experiments, and, to further strengthen the biological significance of the results, also in cells from 5 additional ACM patients and 3 additional controls. Validation was thus performed in RNA samples from 8 ACM patients and 5 control individuals (see Table 1 for clinical information of the individuals).

**Table 2:** ACM-specific miRNAs

	p_adj_	M	ΔC_q ACM_	ΔC_q CTRL_	p_val_	M_val_
hsa-miR-520c-3p	0.012	6.8	29.9	36.7	–	–
hsa-miR-29b-3p	0.020	3.0	28.6	31.6	2.0	0.047
hsa-miR-1183	0.040	4.0	30.7	34.7	n.a.	n.a.

*p*_*adj*_: p-value adjusted for multiple hypothesis testing assessing the significance of differential expression. *M*: log_2_ fold-change value. ΔC_q ACM_ and ΔC_q CTRL_: normalized average expression estimates in ACM and control samples. *p*_*val*_ and *M*_*val*_: p- and M-values from the validation experiments. *n.a.*: test not performed

Differential expression was confirmed for miR-29b-3p but failed for miR-520c-3p, whose expression was below the detection levels in all tested samples (Table 2).

To identify biological pathways being potentially influenced by the differential expression of the identified miRNAs, we next performed Gene Ontology and Reactome pathway enrichment analyses on their target genes. We defined target genes using miRTarBase [20] and, by restricting to only high confidence target genes, identified 84 genes (77 target genes for miR-29b-3p and 7 for miR-520c-3p, no target gene was reported for miR-1183). These were tested for enrichment in Reactome pathways against a background gene set consisting of the 1486 validated target genes of all 208 expressed miRNAs and pathways related to extracellular matrix organization, cell-matrix adhesion and collagen formation were identified (Table 3). The results from the GO enrichment analysis for biological process, cellular component and molecular function supported this finding (Tables S1-S3 in Additional file 2).

**Table 3:** Reactome pathway enrichment analysis results of miRNA target genes

Pathway name	p_adj_	Count	Size	A	B
Extracellular matrix organization	0.000	32	82	30	2
Assembly of collagen fibrils and other multimeric structures	0.000	14	22	14	0
Collagen formation	0.000	15	26	15	0
ECM proteoglycans	0.000	12	20	11	1
Non-integrin membrane-ECM interactions	0.000	13	25	13	0
Degradation of the extracellular matrix	0.000	16	42	15	1
Integrin cell surface interactions	0.000	14	33	13	1
Collagen biosynthesis and modifying enzymes	0.000	9	16	9	0
Platelet degranulation	0.001	11	29	10	1
Response to elevated platelet cytosolic Ca2+	0.001	11	29	10	1
Collagen degradation	0.002	10	24	10	0
Platelet activation, signaling and aggregation	0.006	16	67	15	1
Elastic fibre formation	0.009	8	17	8	0
Laminin interactions	0.011	6	9	6	0
Collagen chain trimerization	0.012	7	13	7	0
Crosslinking of collagen fibrils	0.014	5	6	5	0
NCAM1 interactions	0.046	5	7	5	0

Column *p*_*adj*_: p-value adjusted for multiple hypothesis testing using the method from Bonferroni assessing the significance of enrichment, *Count* and *Size*: number of target genes of ACM-miRNAs or of all detectable miRNAs associated with the pathway. Columns *A* and *B* contain the number of target genes of miR-29b-3p and miR-520c-3p

Since these results based mainly on target genes of miR-29b-3p, we repeated the analysis considering also target genes with lower supporting evidence and thus increased the number of target genes from the other two miRNAs in the analysis (Additional file 2: Table S4). The results from this analysis were comparable (Additional file 2: Tables S6-S9).

Summarizing, we identified 3 miRNAs (miR-520c-3p, miR-29b-3p and miR-1183) to be differentially expressed between ACM patient-derived and control CStCs and confirmed the de-regulation of miR-29b-3p in an extended data set including samples from in total 8 ACM patients and 5 controls. We also found evidence for a potential contribution of miR-29b-3p to ACM development by targeting proteins involved in extracellular matrix organization.

### Genes differentially expressed between ACM and control samples

**Fig. 2:**
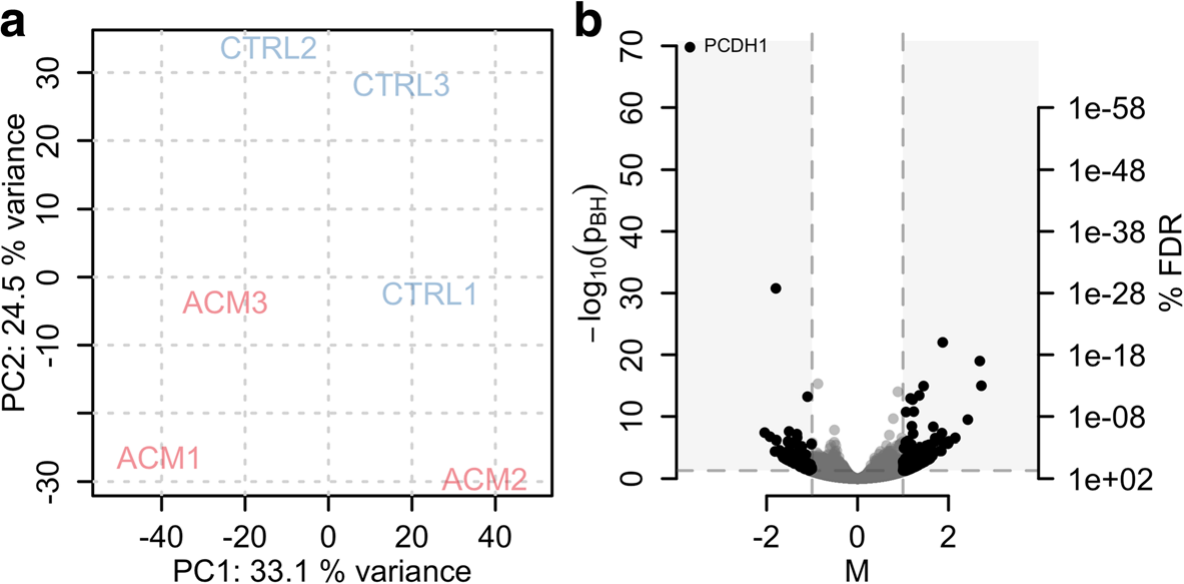
mRNA expression profiling. **a**: PCA results representing the grouping of samples based on their whole genome gene expression profile. **b**: Volcano plot showing the extent (M-value, x-axis) and significance (−log_10_ of Benjamini and Hochberg adjusted *p*-values p_adj_, y-axis) of differential expression between ACM and control samples for each gene. The dashed horizontal and vertical lines represent the cut-off values used to define differentially expressed genes

In order to delineate differences in the gene expression profiles between ACM and control CStCs, we subjected poly-A enriched RNA from 3 ACM and 3 control samples from different individuals to high throughput sequencing for whole genome expression analysis. Gene expression quantification was performed by counting the reads completely aligned within the exon boundaries of any transcript. Most of these reads were assigned to protein coding genes but a considerable number was also assigned to genes from other biotypes such as lincRNAs or snRNAs, allowing quantification of some non-coding genes in the present experiments too (see Figure S1 in Additional file 2 for an overview of read counts per gene biotype). A principal component analysis (PCA) performed on the gene expression profiles showed the expected separation of ACM and CTRL samples (Fig. 2a), but did also suggest the presence of an unknown confounding factor represented by PC1. Including a variable describing the above factor into the linear model and thus alleviating for its potential influence, we performed gene-wise tests for differential expression and identified 272 genes more than two-fold differentially expressed at a 5% FDR (i.e. with an p_adj_ < 0.05; Fig. 2b and Table S10 in Additional file 2; Additional file 3 for the complete results).

For validation, we selected 4 significantly differentially expressed genes (*PCDH1*, *SAXO2*, *SEMA3D*, *NEDD9*). These genes were selected because they were among the most significant differentially expressed genes with a previously described relation to cardiac diseases. For validation of the RNA-seq results we assessed their differential expression in RNA samples from the same individuals, but from cells from different passages and experiments (to ensure the results being independent of the passage/experiment). Regulation of all 4 genes was reconfirmed (Additional file 2: Table S11). To test and validate the biological significance of their regulation in the context of ACM, we evaluated differential expression of the genes in an extended data set consisting of RNA from CStCs derived from 7 ACM patients and 7 control individuals (Table 4).

**Table 4:** Validation of gene regulations in an extended data set

Gene name	p_seq_	M_seq_	p_pcr_	M_pcr_
*PCDH1*	0.00	−3.69	0.13	−1.42
*SAXO2*	0.00	2.72	0.03	2.42
*SEMA3D*	0.00	1.89	0.28	1.08
*NEDD9*	0.00	−1.57	0.01	−1.32

Columns *p*_*seq*_ and *M*_*seq*_: p-value adjusted for multiple hypothesis testing and log_2_ fold change in expression from the RNA-seq experiment, *p*_*pcr*_ and *M*_*pcr*_: *p*-value and log_2_ fold change in expression from the RT-qPCR experiment. The RT-qPCR measurements were conducted in RNA samples from CStCs from 7 ACM patients and 7 control individuals

Regulation of *SAXO2* and *NEDD9* was reconfirmed also on the larger data set, while *PCDH1* and *SEMA3D*, despite being on average more than two-fold, did not reach significance levels.

Subsequent Gene Ontology enrichment analyses of the differentially expressed genes revealed significant enrichment in cellular component terms “integral component of plasma membrane” and “intrinsic component of membrane” (Additional file 2: Table S12) and biological process terms “dopamine transport” and “catecholamine secretion” (Additional file 2: Table S13). Together, these results indicate an enrichment of membrane associated genes among the identified differentially expressed genes.

We next evaluated the expression of genes with ACM-associated variants [31] and, with the exception of JUP, did not find any of these genes being differentially expressed (Additional file 2: Table S14). Interestingly, five of them, *CTNNA3*, *DES*, *DSC2*, *PLN* and *RYR2*, were not expressed, or expressed below the detection limit, in any of the analyzed samples. From the remaining genes, 4 were highly expressed (*LMNA*, *DSP*, *TMEM43* and *JUP*), and 4 moderately (*TTN*, *TGFB3*, *DSG2* and *PKP2*). Expression of *JUP* was highly variable between within-group samples and was on average higher in ACM than in CTRL samples. This was reconfirmed by real-time RT-qPCR measurements in the same RNA (Additional file 2: Figure S2). The observed differential expression did however disappear if additional samples were considered (Additional file 2: Figure S2). Thus, in line with previous reports [8], no biologically significant differential expression of ACM associated genes could be observed on mRNA levels.

Taken together, the results from the gene expression analysis are in agreement with the miRNA target gene analysis, indicating that an integral part of the deregulated genes are functional in the outer cell membrane.

### Combined miRNA and gene expression analysis

Given that some miRNAs are known to repress their target genes mainly by destabilizing their mRNA [32], we combined the miRNA and gene expression data set to evaluate whether a downregulation of target genes of the identified ACM-specific miRNAs is already observable on mRNA levels. Indeed, the average log_2_ fold-change values (M-values) of target genes for most miRNAs were slightly negative, representing an on average lower expression of these genes in ACM than in control samples (Additional file 2: Tables S16-S17). To test whether this observed repression was larger than expected by chance, we compared it to average M-values of same-sized sets of randomly selected target genes of expressed miRNAs. The resulting *p*-values from these tests are listed in Table 5. Of these, the average M-values of miR-29b-3p target genes and the combination of all of the miRNAs’ target genes were significant suggesting that the identified miRNAs are functional and that a tendency of repression is already detectable on mRNA levels.

**Table 5:** Repression of target genes of ACM-specific miRNAs in ACM samples on mRNA levels

miRNA	Average M	p_t_	p_perm_	Gene count
miR-29b-3p	−0.10	0.01	0.03	66
miR-520c-3p	−0.09	0.12	0.28	6
All	−0.10	0.01	0.03	72

*Average M*: average log_2_ fold-change of all target genes of the miRNA, *p*_*t*_: p-value from a Student’s t-test testing the alternative hypothesis of an average M-value < 0, *p*_*perm*_: *p*-value from the permutation test and *Gene count*: number of validate target genes considered in the analysis

Most human miRNAs are encoded in introns of protein coding genes and share in many instances the primary transcription unit with these [33]. Using our mirhostgenes R-package we identified potential host genes for 2 of the ACM-specific miRNAs: the two lincRNAs *C1orf132* and AC058791.1 for miR-29b-3p and the protein coding gene *SP4* for miR-1183. While none of these were significantly differentially expressed, they all showed the expected trend of a higher expression in ACM samples (Additional file 2: Table S18). According to the protein expression atlas [26], *SP4* is expressed in heart muscle tissue. No protein expression data is available for the other two as they are non-coding. According to the Ensembl Expression Atlas however, also *C1orf132* is highly expressed in heart tissue. These miRNAs might therefore, in addition to CStCs, also be expressed in other cell types of the heart.

In conclusion, our analysis on the expression of the miRNAs’ targets genes on mRNA levels suggests the identified miRNAs being functional in the analyzed cells which strengthens the evidence for their potential involvement in ACM.

### Gene network analysis

**Fig. 3:**
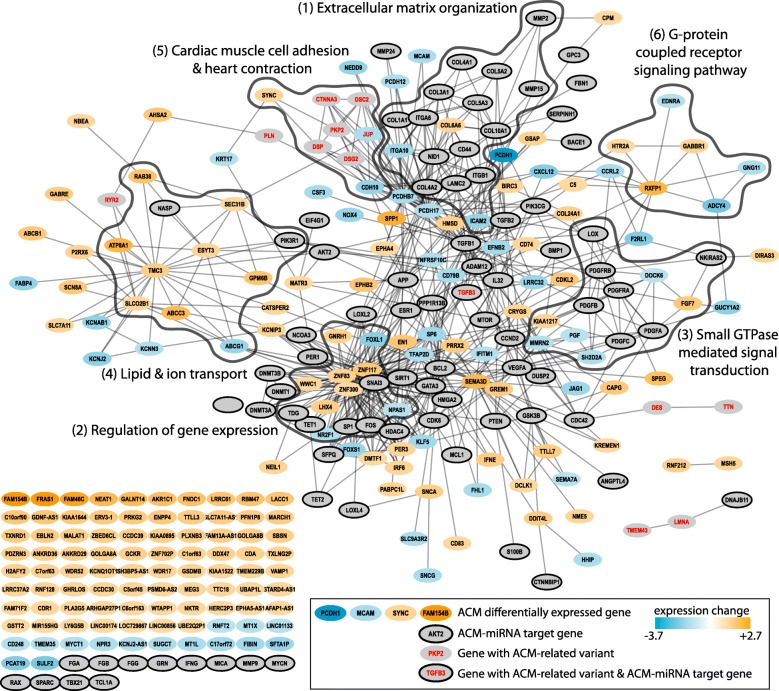
Functional network of differentially expressed genes, CStCs-ACM-miRNA target genes and genes with known ACM-related variants. Nodes represent genes, labeled with their official HGNC symbol, edges functional similarities between genes. Black lines indicate functional modules that represent gene clusters and their common functional annotation

We next performed gene network analyses to evaluate relations between regulated genes, target genes of differentially expressed miRNAs and genes with known ACM-related genetic variants. Such networks allow to identify groups of input gene products that physically interact, that are part of common protein complexes, or that are functionally related. We built a first network based on functional annotations (functional network) of the genes mentioned above. Connected nodes in this network represent thus genes implicated in common biological processes. Clusters of highly connected components were identified and biological processes characteristic for each cluster were determined. Overlapping clusters were further combined resulting in a total of 6 functional modules (FM; resulting network model shown in Fig. 3; see Additional file 4 for module details). The largest, FM_1, consisted of a large number of miRNA targets that are implicated in extracellular matrix organization. Module FM_2 contained multiple transcription factors that are up- or down-regulated in ACM. Module FM_4 included the largest number of differentially expressed genes (9), four of which encoding lipid transport proteins (*ABCC3*, *ATP8A1*, *ESYT3* and *ABCG1*). Genes in FM_5 are related to cardiac muscle cell adhesion and most of them encode desmosomal proteins. Modules FM_3 and FM_6 contained mostly genes involved in signaling processes (small GTPase mediated signal transduction for FM_3 and G-protein coupled receptor signaling for FM_6).

A second gene network was generated using functional relationships from STRING and protein interactions from BioPlex and mentha (combined network). For this analysis we included all genes with a valid read count in the present dataset or which, according to the protein atlas [26], are expressed in the heart tissue. Highly connected clusters of genes were identified as above and those with more than one differentially expressed gene, ACM-miRNA target gene or gene with ACM-related variants were further investigated. The full results are shown online at https://gemex.eurac.edu/bioinf/acm/ (“Clusters in full networks”). Some of the identified clusters showed considerable overlap with the previously identified functional modules, such as c0515 that overlaps FM_5, or the collagen genes containing cluster c0502 resembling FM_1. Additional promising clusters were identified too, like the cell adhesion related cluster c0997 that contained two genes from the protocadherin family (*PCDH12* and *PCDH17*), both being lower expressed in ACM than in control samples, or cluster c0065 that links the differentially expressed gene *ITGA10* with the ACM-miRNA target gene *ITGB1* and with all of its 10 genes being also involved in extracellular matrix organization. Another interesting cluster (c0703) consisted of 16 genes, 6 of which were related to cell adhesion. From these genes, two, the ephrin receptors *EPHA4* and *EPHB2*, were up-regulated and one, the *EFNB* class ephrin ligand *EFNB2*, was down-regulated in ACM cells (Additional file 2: Figure S3).

Summarizing, the functional networks provide an overview of the main biological processes in which both the ACM differentially expressed genes and the ACM-specific miRNA targets play a role. The results indicate that, in addition to cell adhesion related processes, deregulated genes are also involved in lipid transport, inflammation and fibrosis related processes.

## Discussion

In this study, we performed for the first time a comprehensive characterization of the coding and non-coding transcriptome of cardiac stromal cells derived from ACM patients and control individuals. Expression studies in ACM have been performed in the past [9–11], but these were either focused on protein coding genes or on miRNAs and were conducted on RNA extracted directly from heart tissue biopsies or even from explanted hearts. Of the 199 deregulated genes from Gaertner et al. [9] we could detect 191 in our samples, but none of them were differentially expressed. Similarly, none of the 3 genes reported by Akdis et al. [10] were deregulated in our samples. Gaertner et al. performed expression profiling in RNA extracted directly from explanted (final stage) ACM- or non-failing hearts, Akdis et al. in RNA from biopsies, that were even reported to be inhomogeneous in the original article [10]. Missing concordance between studies can thus most likely be attributed to the different sample types being studied, different cell types, or to inhomogeneous cell populations. By performing all analyses in cells of the same type we ensured the results being independent of cell type or tissue-specific differences, limiting the validity of our conclusions however to the cardiac stromal cells analyzed. Recently, 28 miRNAs have been described to be differentially expressed in mouse cardiac muscle cells (HL-1) after shRNA-mediated *PKP2* knock-down [34]. We evaluated the expression for the human equivalents for 12 of these miRNAs in our CStCs. Only two of them, miR-671-3p and miR-487b-3p showed a tendency of differential expression, albeit not reaching significance levels (Additional file 2: Table S15). Also in this case, differences can be explained by different cell types being studied, different species and also the fact that *PKP2* knock-down was artificially induced by shRNA silencing [34].

In our analysis, we identified 3 miRNAs to be differentially expressed between CStCs from ACM and control samples. Two of them (miR-29b and miR-1183) have previously been related to a pathway associated with ACM or other cardiac diseases. miRNAs from the miR-29 family have been shown to play a role in cardiac fibrosis after myocardial infarction targeting collagen, fibrillin and elastin genes [35]. Recently, also desmocollin-2 (*DSC2*), was identified as a direct target of miR-29b in mouse keratinocytes [36], suggesting a potential direct involvement of this miRNA in ACM. miR-1183 on the other hand was identified as a putative serum biomarker for rheumatic heart disease [37]. Analyses on the validated target genes of the ACM-specific miRNAs revealed their involvement in ACM related pathways, such as “extracellular matrix organization”. These results, along with the literature mentioned above, provide evidence for a potential prominent role of miRNAs in the ACM pathogenesis or phenotype maintenance driven by CStCs.

Among the significantly differentially expressed genes were also some potentially interesting candidates: *PCDH1*, the strongest differentially expressed gene, highly expressed in control and very low in ACM samples, encodes the protocadherin 1 membrane protein. Elevated expression of *PCDH1* was found to be related to higher cell aggregation activity [38]. Among the strongest up-regulated genes in ACM was also *SEMA3D* which has been implicated in vascular development and which can inhibit cell motility and tubulogenesis [39]. *PCDH1* and *SEMA3D* were however not de-regulated in all tested cases of the extended validation set, possibly due to a different genetic background of the analyzed ACM cases, and suggesting that different pathways might contribute to ACM in its different subtypes. Another interesting differentially expressed gene was *NEDD9*, a focal adhesion protein that acts as a scaffold to regulate signaling complexes important in cell attachment, migration and invasion [40]. Fibroblasts from *NEDD9* −/− mice showed significantly decreased adhesion strength [41] while over-expression of *NEDD9* was shown to enhance cell speed and haptotaxis towards fibronectin [42]. *NEDD9* is also a validated target gene of the ACM-specific miRNA miR-29-3p and of miR-18a-5p, a miRNA also more than two-fold up-regulated in ACM which regulation did however not reach significance levels (adjusted *p*-value = 0.08). Its more than two-fold down-regulation might hence be mediated or consolidated by these miRNAs.

In analyzing the expression of genes with ACM-associated variants we found that five of the 13 analyzed genes, namely *CTNNA3*, *DES*, *DSC2*, *PLN* and *RYR2*, were not expressed. Therefore, potential pathogenic effects of mutations in those genes is unlikely to be mediated by CStCs.

By evaluating the expression of target genes from the ACM-specific miRNAs we found them to be significantly, albeit only slightly, lower expressed in ACM samples. This moderate downregulation was not completely unexpected given the comparably low expression of the miRNAs and the current opinion of miRNAs acting predominantly through fine-tuning gene expression [43, 44].

The functional network analysis resulted in some interesting clusters of related genes. Among these was the functional module FM_4 containing 9 differentially expressed genes, four of which encode lipid transport proteins. These proteins might play a role in the lipid accumulation described in ACM CStCs. Genes from two other modules, FM_1 containing collagen encoding genes and FM_6 inflammatory response related genes, might be involved in the molecular events leading to fibrosis in ACM. Regarding the analysis of the combined network, an interesting cluster was c0703, a cluster consisting of 16 genes with 13 being involved in ephrin receptor signaling. The membrane bound Eph receptors and ligands are involved in a wide array of developmental processes including cardiovascular, with one of their most prominent biological functions being the modulation of cell adhesion [45]. EphB receptors and ephrins are part of the genetic program activated by β–catenin/Wnt signaling [46]. Eph-ephrin signaling has also been implicated in cardiac stem cell migration into injured tissue after myocardial infarction [47] and has even been proposed as new therapeutic target for that disease [48]. Also an involvement in the modulation of electrical coupling between cardiomyocytes has been reported [49].

### Limitations of the study

At last some limitations of the present study have to be acknowledged. The results presented here base on cultured cells and may hence not be directly expanded to CStCs in their original microenvironment. Different mutations in desmosomal genes might also have an impact on the expression profiles of the cells. Also, it is currently unclear if ACM pathogenesis and disease progression is variant-specific or caused by a common canonical pathway. The small numbers of available ACM cases without any or with common mutations precluded however such mutation-specific differential expression analyses in the present data set. In addition, individuals in the present study do not share a similar genotype and thus any changes possibly identified between samples can not be attributed exclusively to specific variants or sets of variants in specific ACM-related genes. This interesting aspect could however be analyzed in future studies based on samples from individuals with similar genetic background, such as affected and non-affected individuals from the same family.

The small number of biological replicates used in the genome wide screens represent another limitation of the study and hence not all of the results might apply to ACM in general. Nevertheless, we could validate the main findings in a larger set of ACM patients and controls. Still, due to the limited number of samples, the present study should be considered as initial, hypothesis-generating only and further studies evaluating the interconnection between the networks regarding the progression of the disease are needed to fully characterize ACM pathogenesis. Regarding the low number of replicates, it is however also important to highlight that ACM is a rare disease with low incidence in the population [1, 2] and that it is difficult to obtain CStCs from ACM patients due to the small rate of patients that undergo cardiac biopsies.

Selection of appropriate control samples is crucial for any study. All control samples used for transcriptome analysis and miRNA profiling derive from deceased individuals and might thus not be completely identical to biopsies from living hearts. By taking the samples from deceased organ donors without heart disease, which ensures special care to the explanted organs, we tried to minimize any potential biases. CStCs from control samples were also generated using the same protocols and cultured under the same conditions as CStCs from ACM individuals.

Due to the phenotypic overlap of ACM with those of other cardiomyopathies, particularly with the arrhythmic form of idiopathic dilated cardiomyopathy (DCM) [50], we can not completely exclude the miRNA and gene expression patterns presented here being shared across some cardiomyopathies. Recently, genetic overlaps between ACM and DCM have been described: mutations in genes encoding desmosomal proteins have been reported in DCM cases [51] and mutations in *TTN* [50], *PLN* [52] and *FLNC* [53] have been associate with both ACM and DCM. Abnormalities such as epsilon waves and right ventricular dilation, considered classical hallmarks of ACM, have been described recently in patients with Brugada syndrome [54, 55], along with mutations in the *PKP2* gene [56]. We can therefore not exclude that the identified molecular signatures are specific for ACM and do not overlap, at least to a certain degree, with signatures in other primary cardiomyopathies. Still, for the potential overlap with DCM, it has to be noted that the shared phenotypic and structural overlap of both diseases is seen in end-stage heart failures, while the results in this study have been performed in early-stage ACM patients.

Finally, all of the analyzed individuals in this study are male, and some conclusions or results might thus not be applicable to female ACM patients. Despite the lower prevalence of ACM in females, it would however be of utmost interest, also in the light of gender medicine, to dissect in future studies gender-specific differences in ACM disease development and progression.

## Conclusions

We determined for the first time the coding and non-coding transcriptome of ACM in cardiac stromal cells. We found evidence for a potential prominent role of miRNAs in ACM pathogenesis or phenotype maintenance. Due to the small sample sizes used for whole genome expression profiling, the presented results might however not be extended to all cell types involved in ACM or to all different subtypes of ACM. Taken together, the comprehensive data set and the results presented here might serve as a resource for future studies elucidating the role of the identified genes, miRNAs or pathways in ACM.

## Additional files

Additional file 1: miRNA expression results. Contains the complete data and results from the miRNA expression profiling. (XLSX 82 kb)

Additional file 2: Supplementary information. Contains supplementary information, tables and figures. (PDF 1224 kb)

Additional file 3: Gene expression results. Contains gene quantification data and results from the transcriptome profiling. (XLSX 4484 kb)

Additional file 4: Network analysis results. Contains functional module definitions and results from the functional network analysis. (XLSX 17 kb)

## Abbreviations

ACM: Arrhythmogenic cardiomyopathy
CStCs: Cardiac stromal cells
DCM: Dilated cardiomyopathy
FDR: False discovery rate
GO: Gene ontology
PCA: Principal component analysis
TLDA: TaqMan Low Density Arrays;
