# Supplementary material for: The arrhythmogenic cardiomyopathy-specific coding and non-coding transcriptome in human cardiac stromal cells
Source: BMC Genomics. 2018 Jun 25;19:491. doi: 10.1186/s12864-018-4876-6 (PMC6019788; doi:10.1186/s12864-018-4876-6)
Supplement: Supplementary file 2 — Supplementary information. Contains supplementary information, tables and figures. (PDF 1224 kb) [file 12864_2018_4876_MOESM2_ESM.pdf]

Supplementary Information for: **The arrhythmogenic cardiomyopathy-specific coding and non-coding transcriptome in human cardiac stromal cells**

Johannes Rainer[1,*], Viviana Meraviglia[1,*], Hagen Blankenburg[1], Chiara Piubelli[1], Peter P. Pramstaller[1], Adolfo Paolin[2], Elisa Cogliati[2], Giulio Pompilio[3], Elena Sommariva[3], Francisco S. Domingues[1], Alessandra Rossini[1]

1: Institute for Biomedicine, Eurac Research, Viale Druso 1, 39100 Bolzano, Italy.

2: Treviso Tissue Bank Foundation, Piazzalo Ospedale 1, 31100, Treviso, Italy.

3: Vascular Biology and Regenerative Medicine Unit, Centro Cardiologico Monzino IRCCS, via Parea 4, Milan 20138, Italy.

*: Corresponding author

Contents

1 Extended Material and Methods **2**
1.1 RT-qPCR primers 2

2 miRNA target gene analysis **2**
2.1 High confidence validated targets 2
2.2 All validated target genes 3

3 mRNA expression profiling **6**

4 Combined miRNA-mRNA expression analysis **13**

5 Network analysis **16**

1 Extended Material and Methods

1.1 RT-qPCR primers

TaqMan Gene Expression Assays were used to measure PCDH1 (Hs00170174_m1), SAXO2 (Hs04184626_m1), SEMA3D (Hs01008854_m1), NEDD9 (Hs00610590_m1) and TBP (Hs00427620_m1) following manufacturer's instructions. To validate TLDA-derived miRNA expression data, individual single-assay primers and target probes (Applied Biosystems) for miR-28-3p (#002446), miR-29b-3p (#000413) and miR-520c-3p (#002400) were used.

2 miRNA target gene analysis

2.1 High confidence validated targets

	p_{adj}	Count	Size	Term	A	B
GO:0044259	0.00	15	37	multicellular organismal macromolecule metabolic process	15	0
GO:0030574	0.00	11	23	collagen catabolic process	11	0
GO:0002576	0.00	11	29	platelet degranulation	10	1
GO:0007160	0.01	9	23	cell-matrix adhesion	8	1
GO:0006304	0.01	9	23	DNA modification	9	0
GO:0030198	0.01	10	37	extracellular matrix organization	9	1
GO:0071230	0.04	9	27	cellular response to amino acid stimulus	8	1
GO:0030199	0.04	8	21	collagen fibril organization	8	0
GO:2000587	0.05	4	4	negative regulation of platelet-derived growth factor receptor-beta signaling pathway	4	0

Supplementary Table S1: Biological process GO-terms enriched with high confidence validated target genes of the significantly differentially expressed miRNAs: hsa-miR-29b-3p and hsa-miR-520c-3p (hsa-miR-1183 does not have any validated target genes). Column p_{adj} contains the Bonferroni-adjusted p-values assessing the significance of enrichment, columns *Count* and *Size* the number of validated target genes of regulated miRNAs and of all detectable miRNAs associated with the respective term. Columns *A* and *B* list the number of validated target genes of miR-29b-3p and miR-520c-3p, miR-324-5p annotated to the term.

	p_{adj}	Count	Size	Term	A	B
GO:0005788	0.00	13	33	endoplasmic reticulum lumen	13	0
GO:0031093	0.00	11	23	platelet alpha granule lumen	10	1
GO:0005615	0.00	29	189	extracellular space	26	3
GO:0098644	0.00	6	9	complex of collagen trimers	6	0
GO:0005578	0.01	10	41	proteinaceous extracellular matrix	9	1
GO:0060205	0.01	13	61	cytoplasmic vesicle lumen	12	1

Supplementary Table S2: Cellular component GO-terms enriched with high confidence validated target genes of the significantly differentially expressed miRNAs: hsa-miR-29b-3p and hsa-miR-520c-3p (hsa-miR-1183 does not have any validated target genes). Column p_{adj} contains the Bonferroni-adjusted p-values assessing the significance of enrichment, columns *Count* and *Size* the number of validated target genes of regulated miRNAs and of all detectable miRNAs associated with the respective term. Columns *A* and *B* list the number of validated target genes of miR-29b-3p and miR-520c-3p, miR-324-5p annotated to the term.

	p_{adj}	Count	Size	Term	A	B
GO:0048407	0.00	7	9	platelet-derived growth factor binding	7	0
GO:0005161	0.00	7	10	platelet-derived growth factor receptor binding	7	0
GO:0005518	0.00	9	21	collagen binding	8	1

Supplementary Table S3: Molecular function GO-terms enriched with high confidence validated target genes of the significantly differentially expressed miRNAs: hsa-miR-29b-3p and hsa-miR-520c-3p (hsa-miR-1183 does not have any validated target genes). Column p_{adj} contains the Bonferroni-adjusted p-values assessing the significance of enrichment, columns *Count* and *Size* the number of validated target genes of regulated miRNAs and of all detectable miRNAs associated with the respective term. Columns *A* and *B* list the number of validated target genes of miR-29b-3p and miR-520c-3p, miR-324-5p annotated to the term.

2.2 All validated target genes

We conducted the same enrichment analysis on all validated target genes from the miRTarBase with evidence *Functional MTI* and *Functional MTI (Weak)*. Compared to the analysis in the previous section, this adds mostly target genes that were identified by high throughput methods PAR-CLIP, HITS-CLIP and CLASH. In sum, 703 target genes of the deregulated miRNAs were compared against the background set of 13090 target genes.

	No. genes	Functional MTI	Functional MTI (Weak)	No. publications
hsa-miR-29b-3p	221	77	160	73
hsa-miR-520c-3p	423	7	417	23
hsa-miR-1183	77	0	77	12

Supplementary Table S4: Summary for the miRNA target gene interactions with *Functional MTI* support type and *Functional MTI (Weak)* defined in the miRTarBase for the miRNAs significantly differentially expressed between ACM and control samples. Shown are the number of validated target genes for each miRNA, the number of genes for each evidence grade type and the number of publications in which the interactions were described.

Experiment type	No. MTIs validated
PAR-CLIP	563
HITS-CLIP	262
Luciferase reporter assay	107
qRT-PCR	90
Western blot	68
Microarray	37
CLASH	26
Immunohistochemistry	9
Immunofluorescence	6
Northern blot	6
ELISA	5
Flow	3
GFP reporter assay	3
Immunoblot	3
Reporter assay;Other	3
Immunoprecipitaion	2
Reporter assay	2
Western blot	1
ChIP	1
QRTPCR	1
Reporter assay;Western blot;Other	1
Reporter assay;Western blot;qRT-PCR	1
Western blot;Microarray;Other	1

Supplementary Table S5: Table listing the experiments that have been performed to validate the miRNA target gene interaction (MTI). Shown are the experimental procedures as described in the original publication as well as the number of MTIs validated with the respective method; in some instances the MTI was evaluated with more than one experiment.

Pathway name	p_{adj}	Count	Size	A	B	C
Extracellular matrix organization	0.00	42	226	36	6	0
Assembly of collagen fibrils and other multimeric structures	0.00	19	51	19	0	0
Collagen formation	0.00	21	73	21	0	0
Non-integrin membrane-ECM interactions	0.00	18	54	16	2	0
Integrin cell surface interactions	0.00	19	69	17	2	0
Collagen degradation	0.00	16	51	15	1	0
ECM proteoglycans	0.00	16	57	14	2	0
Degradation of the extracellular matrix	0.00	22	105	20	2	0
Anchoring fibril formation	0.00	8	13	8	0	0
Collagen biosynthesis and modifying enzymes	0.00	15	52	15	0	0
Collagen chain trimerization	0.00	12	33	12	0	0
Laminin interactions	0.00	10	26	9	1	0
Response to elevated platelet cytosolic Ca2+	0.01	19	102	13	5	1
Platelet degranulation	0.02	18	97	13	4	1
Hemostasis	0.02	51	473	24	24	5
Platelet activation, signaling and aggregation	0.02	29	210	18	9	3
TET1,2,3 and TDG demethylate DNA	0.02	4	4	4	0	0
Signaling by PDGF	0.04	37	311	22	14	4
CD28 co-stimulation	0.05	9	29	5	3	2

Continued on next page

Continued from previous page

Pathway name	p_{adj}	Count	Size	A	B	C

Supplementary Table S6: Reactome pathways enriched with validated target genes of the significantly differentially expressed miRNAs: hsa-miR-29b-3p, hsa-miR-520c-3p and hsa-miR-1183. Column p_{adj} contains the Bonferroni-adjusted p-values assessing the significance of enrichment, columns *Count* and *Size* the number of validated target genes of regulated miRNAs and of all detectable miRNAs associated with the respective pathway. Columns *A*, *B* and *C* list the number of validated target genes of miR-29b-3p, miR-520c-3p and miR-1183 annotated to the pathway, respectively.

	p_{adj}	Count	Size	Term	A	B	C
GO:0044259	0.00	25	91	multicellular organismal macromolecule metabolic process	23	2	0
GO:0030574	0.00	16	50	collagen catabolic process	16	0	0
GO:1990314	0.00	6	7	cellular response to insulin-like growth factor stimulus	3	3	1
GO:0030199	0.01	10	29	collagen fibril organization	9	1	0

Supplementary Table S7: Biological process GO terms enriched with validated target genes of the significantly differentially expressed miRNAs: hsa-miR-29b-3p, hsa-miR-520c-3p and hsa-miR-1183. Column p_{adj} contains the Bonferroni-adjusted p-values assessing the significance of enrichment, columns *Count* and *Size* the number of validated target genes of regulated miRNAs and of all detectable miRNAs associated with the respective term. Columns *A*, *B* and *C* list the number of validated target genes of miR-29b-3p, miR-520c-3p and miR-1183 annotated to the term, respectively.

	p_{adj}	Count	Size	Term	A	B	C
GO:0098644	0.00	10	16	complex of collagen trimers	10	0	0
GO:0031974	0.00	276	4039	membrane-enclosed lumen	110	143	33
GO:0031012	0.01	42	379	extracellular matrix	35	6	1
GO:0005788	0.01	24	165	endoplasmic reticulum lumen	20	3	1
GO:0031093	0.01	12	50	platelet alpha granule lumen	10	2	0
GO:0005581	0.02	11	47	collagen trimer	11	0	0
GO:0005587	0.03	4	5	collagen type IV trimer	4	0	0

Supplementary Table S8: Cellular component GO terms enriched with validated target genes of the significantly differentially expressed miRNAs: hsa-miR-29b-3p, hsa-miR-520c-3p and hsa-miR-1183. Column p_{adj} contains the Bonferroni-adjusted p-values assessing the significance of enrichment, columns *Count* and *Size* the number of validated target genes of regulated miRNAs and of all detectable miRNAs associated with the respective term. Columns *A*, *B* and *C* list the number of validated target genes of miR-29b-3p, miR-520c-3p and miR-1183 annotated to the term, respectively.

	p_{adj}	Count	Size	Term	A	B	C
GO:0048407	0.00	8	11	platelet-derived growth factor binding	8	0	0
GO:0005161	0.01	7	15	platelet-derived growth factor receptor binding	7	0	0

Supplementary Table S9: Molecular function GO terms enriched with validated target genes of the significantly differentially expressed miRNAs: hsa-miR-29b-3p, hsa-miR-520c-3p and hsa-miR-1183. Column p_{adj} contains the Bonferroni-adjusted p-values assessing the significance of enrichment, columns *Count* and *Size* the number of validated target genes of regulated miRNAs and of all detectable miRNAs associated with the respective term. Columns *A*, *B* and *C* list the number of validated target genes of miR-29b-3p, miR-520c-3p and miR-1183 annotated to the term, respectively.

3 mRNA expression profiling

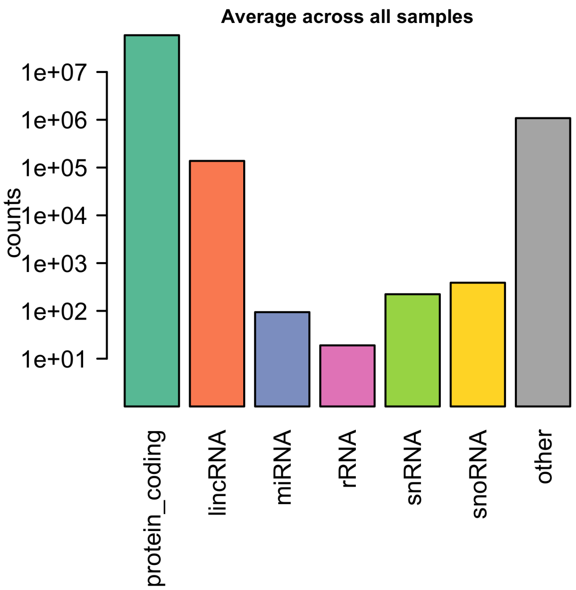

Supplementary Figure S1: Average numer of reads across all samples assigned to genes from different biotypes (as defined in the Ensembl database version 81).

	Gene ID	Gene name	Biotype	p_{adj}	M	ACM_{count}	$CTRL_{count}$
1	ENSG00000156453	PCDH1	6	0.000	-3.69	239.570	2593.64
2	ENSG00000164251	F2RL1	6	0.000	-1.80	4207.333	9927.10
3	ENSG00000245532	NEAT1	2	0.000	1.87	15515.161	3718.58
4	ENSG00000138759	FRAS1	6	0.000	2.69	1025.281	149.01
5	ENSG00000188659	SAXO2	6	0.000	2.72	212.147	15.07
6	ENSG00000173209	AHSA2	6	0.000	1.45	1196.262	378.81
7	ENSG00000214021	TTLL3	6	0.000	1.36	741.337	264.63
8	ENSG00000126950	TMEM35	6	0.000	-1.10	1291.674	3305.59
9	ENSG00000204681	GABBR1	6	0.000	1.16	1279.182	514.69
10	ENSG00000167766	ZNF83	6	0.000	1.21	1540.002	563.25
11	ENSG00000175265	GOLGA8A	6	0.000	1.24	1166.140	383.45
12	ENSG00000135164	DMTF1	6	0.000	1.07	2492.562	1059.42
13	ENSG00000183508	FAM46C	6	0.000	2.43	162.435	15.14
14	ENSG00000099284	H2AFY2	6	0.000	1.19	801.330	344.72
15	ENSG00000072195	SPEG	6	0.000	1.67	1580.742	523.59
16	ENSG00000160179	ABCG1	6	0.000	-1.51	371.120	868.81
17	ENSG00000129467	ADCY4	6	0.000	-2.04	48.184	263.91
18	ENSG00000189398	OR7E12P	14	0.000	1.86	159.494	24.70
19	ENSG00000117616	RSRP1	6	0.000	1.22	2074.737	800.15
20	ENSG00000250786	SNHG18	2	0.000	-1.33	753.289	1756.79
21	ENSG00000196562	SULF2	6	0.000	-1.93	571.992	2233.81
22	ENSG00000125266	EFNB2	6	0.000	-1.33	534.936	1092.19
23	ENSG00000118785	SPP1	6	0.000	2.15	101.860	7.72
24	ENSG00000232931	LINC00342	2	0.000	1.71	472.763	94.23
25	ENSG00000107562	CXCL12	6	0.000	-1.79	8661.636	39734.77
26	ENSG00000161010	C5orf45	6	0.000	1.11	1140.607	489.58
27	ENSG00000171509	RXFP1	6	0.000	2.01	157.896	20.98
28	ENSG00000040731	CDH10	6	0.000	-1.53	334.185	779.69
29	ENSG00000187134	AKR1C1	6	0.000	1.70	18796.918	4822.61
30	ENSG00000114857	NKTR	6	0.000	1.06	4782.672	1814.80
31	ENSG00000153993	SEMA3D	6	0.000	1.89	3732.597	470.43
32	ENSG00000101384	JAG1	6	0.000	-1.45	5420.422	11822.52
33	ENSG00000124406	ATP8A1	6	0.000	1.99	932.756	54.57
34	ENSG00000257219	RP11-54A9.1	2	0.000	-1.01	803.574	1746.72
35	ENSG00000101104	PABPC1L	6	0.000	1.18	565.077	236.88
36	ENSG00000163694	RBM47	6	0.000	1.57	339.731	100.36
37	ENSG00000179630	LACC1	6	0.000	1.51	3077.724	842.66
38	ENSG00000108342	CSF3	6	0.000	-1.28	2137.497	3786.67
39	ENSG00000164161	HHIP	6	0.000	-1.23	35321.685	67046.89
40	ENSG00000137941	TTLL7	6	0.000	1.37	1671.820	562.42
41	ENSG00000264112	RP11-159D12.2	2	0.000	1.51	515.299	112.78
42	ENSG00000259976	RP11-553L6.5	2	0.000	1.02	278.407	117.71
43	ENSG00000271533	RP3-368A4.6	7	0.000	1.51	223.229	45.13
44	ENSG00000042493	CAPG	6	0.000	1.45	1412.717	527.43
45	ENSG00000261490	RP11-448G15.3	8	0.000	1.50	285.285	66.91
46	ENSG00000279759	CTC-425O23.5	9	0.000	1.35	147.656	44.75
47	ENSG00000213462	ERV3-1	6	0.000	1.43	3856.066	933.95
48	ENSG00000247095	MIR210HG	2	0.000	-1.51	80.119	202.58
49	ENSG00000257732	RP11-818F20.5	1	0.000	1.55	117.875	30.75
50	ENSG00000176678	FOXL1	6	0.000	-1.71	782.248	3791.55
51	ENSG00000223745	RP4-717I23.3	5	0.000	1.17	263.361	90.27
52	ENSG00000108846	ABCC3	6	0.000	1.84	1245.472	105.94
53	ENSG00000158089	GALNT14	6	0.000	1.81	152.635	22.80
54	ENSG00000197083	ZNF300P1	12	0.000	1.65	109.516	23.60
55	ENSG00000152926	ZNF117	6	0.000	1.48	6151.888	1015.78
56	ENSG00000267107	PCAT19	2	0.000	-1.82	1.420	43.41
57	ENSG00000198431	TXNRD1	6	0.000	1.32	99509.372	34082.04
58	ENSG00000111859	NEDD9	6	0.000	-1.57	649.337	2236.54
59	ENSG00000228492	RAB11FIP1P1	4	0.000	1.66	77.701	12.79
60	ENSG00000138944	KIAA1644	6	0.000	1.44	2413.772	1088.31
61	ENSG00000143127	ITGA10	6	0.000	-1.33	369.846	1433.92
62	ENSG00000246451	RP11-894P9.1	1	0.000	1.17	270.907	99.75
63	ENSG00000102554	KLF5	6	0.000	-1.38	532.710	1401.53

Continued on next page

Continued from previous page

	Gene ID	Gene name	Biotype	p_{adj}	M	ACM_{count}	$CTRL_{count}$
64	ENSG00000131002	TXLNGY	12	0.000	1.20	2080.032	582.20
65	ENSG00000152402	GUCY1A2	6	0.000	-1.67	78.340	309.52
66	ENSG00000175745	NR2F1	6	0.000	-1.14	1009.562	2472.01
67	ENSG00000164694	FNDC1	6	0.000	1.65	278.187	54.28
68	ENSG00000275993	CH507-42P11.8	6	0.000	-1.55	73.052	292.05
69	ENSG00000154493	C10orf90	6	0.000	1.49	69.718	18.18
70	ENSG00000121440	PDZRN3	6	0.000	1.25	1232.838	581.30
71	ENSG00000174501	ANKRD36C	6	0.000	1.36	430.993	119.12
72	ENSG00000135976	ANKRD36	6	0.000	1.25	379.251	124.91
73	ENSG00000206530	CFAP44	6	0.000	1.19	288.827	80.53
74	ENSG00000261326	LINC01355	2	0.000	1.64	111.795	13.11
75	ENSG00000046653	GPM6B	6	0.000	1.58	68.624	15.95
76	ENSG00000281183	NPTN-IT1	7	0.000	1.44	121.407	27.71
77	ENSG00000273151	RP11-449P15.2	1	0.000	1.24	116.063	35.66
78	ENSG00000166923	GREM1	6	0.000	1.38	53857.991	16469.75
79	ENSG00000086991	NOX4	6	0.000	-1.50	419.887	1074.73
80	ENSG00000123700	KCNJ2	6	0.000	-1.64	453.632	2888.13
81	ENSG00000145358	DDIT4L	6	0.000	1.34	1872.613	578.55
82	ENSG00000108622	ICAM2	6	0.000	-1.53	102.849	369.18
83	ENSG00000227671	RP11-488L18.4	12	0.000	1.07	1234.301	409.21
84	ENSG00000123892	RAB38	6	0.001	1.60	318.937	41.12
85	ENSG00000105792	CFAP69	6	0.001	1.19	167.379	53.97
86	ENSG00000170323	FABP4	6	0.001	-1.59	15.170	165.11
87	ENSG00000271430	RP3-368A4.5	7	0.001	1.05	374.203	133.58
88	ENSG00000238083	LRRC37A2	6	0.001	1.13	198.921	88.92
89	ENSG00000162520	SYNC	6	0.001	1.15	1859.886	850.43
90	ENSG00000169282	KCNAB1	6	0.001	-1.58	81.750	816.11
91	ENSG00000121454	LHX4	6	0.001	1.28	111.766	32.47
92	ENSG00000127399	LRRC61	6	0.001	1.57	286.107	10.51
93	ENSG00000173801	JUP	6	0.001	-1.19	3190.984	6778.65
94	ENSG00000224660	SH3BP5-AS1	1	0.001	1.18	281.674	91.83
95	ENSG00000163064	EN1	6	0.001	1.49	88.952	0.94
96	ENSG00000138769	CDKL2	6	0.001	1.50	75.005	13.08
97	ENSG00000248587	GDNF-AS1	2	0.001	1.49	84.392	13.79
98	ENSG00000265194	RP11-70L8.4	1	0.001	1.51	49.643	8.19
99	ENSG00000215252	GOLGA8B	6	0.001	1.27	1159.118	299.60
100	ENSG00000127920	GNG11	6	0.001	-1.19	12654.749	33801.22
101	ENSG00000225383	SFTA1P	2	0.001	-1.50	91.004	405.26
102	ENSG00000133216	EPHB2	6	0.001	1.55	2703.870	349.71
103	ENSG00000259972	AC009120.6	5	0.001	1.25	132.808	40.49
104	ENSG00000075826	SEC31B	6	0.001	1.06	196.057	75.38
105	ENSG00000179772	FOXS1	6	0.001	-1.53	165.406	1140.62
106	ENSG00000073605	GSDMB	6	0.001	1.15	217.739	78.92
107	ENSG00000259498	RP11-244F12.3	1	0.001	1.32	212.884	43.88
108	ENSG00000167157	PRRX2	6	0.001	1.37	352.361	136.65
109	ENSG00000176971	FIBIN	6	0.001	-1.36	298.451	788.00
110	ENSG00000225855	RUSC1-AS1	1	0.001	1.08	299.289	107.67
111	ENSG00000242779	ZNF702P	10	0.001	1.22	432.156	142.82
112	ENSG00000085563	ABCB1	6	0.001	1.49	133.338	5.03
113	ENSG00000280063	RP11-295D4.3	9	0.001	1.38	91.606	21.09
114	ENSG00000145908	ZNF300	6	0.002	1.00	871.456	325.88
115	ENSG00000137507	LRRC32	6	0.002	-1.47	1303.375	7675.12
116	ENSG00000253522	CTC-231O11.1	2	0.002	1.33	956.134	159.33
117	ENSG00000251562	MALAT1	2	0.002	1.31	6257.242	1382.88
118	ENSG00000263826	RP11-573D15.9	1	0.002	1.28	69.992	19.86
119	ENSG00000120279	MYCT1	6	0.002	-1.13	3084.140	5280.18
120	ENSG00000251194	RP1-68D18.2	7	0.002	1.48	31.561	3.63
121	ENSG00000015479	MATR3	6	0.002	1.08	160.167	54.90
122	ENSG00000196876	SCN8A	6	0.002	1.46	175.771	29.43
123	ENSG00000145075	CCDC39	6	0.002	1.29	130.674	29.38
124	ENSG00000102287	GABRE	6	0.002	1.34	953.624	251.87
125	ENSG00000213782	DDX47	6	0.002	1.22	165.107	49.03
126	ENSG00000230551	CTB-89H12.4	5	0.002	1.04	886.437	298.17

Continued on next page

Continued from previous page

	Gene ID	Gene name	Biotype	p_{adj}	M	ACM_{count}	$CTRL_{count}$
127	ENSG00000270392	PFN1P2	10	0.002	1.34	58.236	13.99
128	ENSG00000140285	FGF7	6	0.002	1.43	868.322	70.68
129	ENSG00000270069	MIR222HG	2	0.003	1.18	691.521	230.30
130	ENSG00000138669	PRKG2	6	0.003	1.42	95.434	20.43
131	ENSG00000173267	SNCG	6	0.003	-1.26	181.274	549.56
132	ENSG00000279227	AC009303.2	9	0.003	-1.11	38.323	84.20
133	ENSG00000001561	ENPP4	6	0.003	1.40	371.919	46.73
134	ENSG00000250397	RP11-1391J7.1	1	0.003	1.35	62.245	14.25
135	ENSG00000189136	UBE2Q2P1	12	0.003	1.01	192.063	66.06
136	ENSG00000151012	SLC7A11	6	0.003	1.17	16951.102	5874.43
137	ENSG00000269352	PTOV1-AS2	1	0.004	1.11	97.713	41.41
138	ENSG00000189120	SP6	6	0.004	-1.39	26.318	139.27
139	ENSG00000260549	MT1L	13	0.004	-1.21	1237.304	3517.18
140	ENSG00000184995	IFNE	6	0.004	1.33	191.062	49.74
141	ENSG00000171502	COL24A1	6	0.004	1.25	101.634	31.75
142	ENSG00000139190	VAMP1	6	0.004	1.13	452.806	153.19
143	ENSG00000224383	PRR29	6	0.004	-1.32	39.159	117.90
144	ENSG00000234883	MIR155HG	2	0.004	1.04	126.999	57.94
145	ENSG00000145416	MARCH1	6	0.004	1.33	152.019	26.34
146	ENSG00000065054	SLC9A3R2	6	0.004	-1.22	2364.209	6878.80
147	ENSG00000204410	MSH5	6	0.004	1.14	110.051	43.14
148	ENSG00000175600	SUGCT	6	0.004	-1.18	944.770	2014.19
149	ENSG00000172915	NBEA	6	0.004	1.06	335.159	163.20
150	ENSG00000186088	GSAP	6	0.004	1.21	414.378	132.73
151	ENSG00000198753	PLXNB3	6	0.005	1.28	763.281	243.30
152	ENSG00000255423	EBLN2	6	0.005	1.32	70.204	13.80
153	ENSG00000259583	RP11-66B24.4	1	0.005	1.15	942.889	225.96
154	ENSG00000130751	NPAS1	6	0.005	-1.04	147.915	361.91
155	ENSG00000140398	NEIL1	6	0.005	1.15	74.666	29.39
156	ENSG00000280927	CTBP1-AS	1	0.005	1.23	68.354	20.28
157	ENSG00000174807	CD248	6	0.006	-1.08	2543.757	6833.54
158	ENSG00000279382	RP11-449J21.3	9	0.006	1.03	92.249	39.91
159	ENSG00000113212	PCDHB7	6	0.006	-1.28	30.432	106.65
160	ENSG00000250033	SLC7A11-AS1	5	0.006	1.34	110.723	15.16
161	ENSG00000205085	FAM71F2	6	0.007	1.08	103.178	36.89
162	ENSG00000147437	GNRH1	6	0.007	1.25	53.286	13.93
163	ENSG00000022267	FHL1	6	0.007	-1.28	2305.341	7152.97
164	ENSG00000084734	GCKR	6	0.007	1.23	298.150	97.38
165	ENSG00000128422	KRT17	6	0.007	-1.12	40.464	89.49
166	ENSG00000236017	ASMTL-AS1	1	0.007	1.25	111.446	29.65
167	ENSG00000279312	RP3-331H24.7	9	0.007	1.28	109.384	23.05
168	ENSG00000164542	KIAA0895	6	0.008	1.29	139.048	27.96
169	ENSG00000145335	SNCA	6	0.008	1.31	184.365	23.67
170	ENSG00000272620	AFAP1-AS1	1	0.008	1.05	82.578	29.03
171	ENSG00000023445	BIRC3	6	0.008	1.11	177.826	58.41
172	ENSG00000272574	RP11-359K18.4	7	0.008	1.24	48.113	11.22
173	ENSG00000188707	ZBED6CL	6	0.008	1.31	109.346	8.97
174	ENSG00000106804	C5	6	0.008	1.01	157.480	78.93
175	ENSG00000279649	RP11-96D1.8	9	0.008	1.16	75.413	22.64
176	ENSG00000187193	MT1X	6	0.008	-1.07	188.337	504.20
177	ENSG00000188869	TMC3	6	0.008	1.29	80.306	6.56
178	ENSG00000121797	CCRL2	6	0.008	-1.26	12.157	42.72
179	ENSG00000269821	KCNQ1OT1	1	0.009	1.18	496.438	115.65
180	ENSG00000007312	CD79B	6	0.009	-1.28	3.166	25.14
181	ENSG00000275131	CH17-472G23.4	14	0.009	1.15	579.285	197.48
182	ENSG00000162522	KIAA1522	6	0.009	1.14	1783.058	784.51
183	ENSG00000221887	HMSD	6	0.009	1.25	47.789	3.87
184	ENSG00000248019	FAM13A-AS1	1	0.010	1.28	39.777	4.51
185	ENSG00000189001	SBSN	6	0.011	1.27	82.302	13.04
186	ENSG00000171658	RP11-443P15.2	12	0.011	1.16	545.089	158.64
187	ENSG00000113389	NPR3	6	0.011	-1.15	424.981	1248.61
188	ENSG00000178222	RNF212	6	0.011	1.27	91.813	12.03
189	ENSG00000008197	TFAP2D	6	0.012	-1.23	1.129	28.24

Continued on next page

Continued from previous page

	Gene ID	Gene name	Biotype	p_{adj}	M	ACM_{count}	$CTRL_{count}$
190	ENSG00000076706	MCAM	6	0.012	-1.26	1966.419	7374.05
191	ENSG00000166762	CATSPER2	6	0.013	1.10	84.253	28.22
192	ENSG00000113555	PCDH12	6	0.013	-1.20	73.346	232.21
193	ENSG00000173535	TNFRSF10C	6	0.013	-1.15	393.389	1015.82
194	ENSG00000273599	RP11-59C5.3	1	0.013	1.08	74.373	27.54
195	ENSG00000173269	MMRN2	6	0.014	-1.10	183.782	462.35
196	ENSG00000154065	ANKRD29	6	0.014	1.24	264.014	47.39
197	ENSG00000246922	UBAP1L	6	0.014	1.08	76.291	26.16
198	ENSG00000259820	AC083843.1	2	0.015	1.16	105.906	27.90
199	ENSG00000232346	SC22CB-1E7.1	4	0.015	-1.05	1241.840	3128.04
200	ENSG00000130158	DOCK6	6	0.015	-1.01	1936.538	4153.77
201	ENSG00000099957	P2RX6	6	0.015	1.20	70.339	18.14
202	ENSG00000156042	CFAP70	6	0.016	1.08	63.902	18.75
203	ENSG00000183762	KREMEN1	6	0.016	1.06	965.627	431.96
204	ENSG00000198133	TMEM229B	6	0.016	1.14	90.211	28.08
205	ENSG00000158825	CDA	6	0.016	1.21	305.554	90.47
206	ENSG00000215769	RP13-104F24.2	5	0.016	1.06	66.276	21.31
207	ENSG00000229152	ANKRD10-IT1	7	0.017	1.16	130.700	27.42
208	ENSG00000123870	ZNF137P	12	0.018	1.01	87.216	33.60
209	ENSG00000269984	RP11-362K14.5	1	0.018	1.15	38.110	10.70
210	ENSG00000115041	KCNIP3	6	0.018	1.20	205.017	52.84
211	ENSG00000150627	WDR17	6	0.020	1.15	63.566	6.12
212	ENSG00000213139	CRYGS	6	0.020	1.16	49.248	12.29
213	ENSG00000259562	RP11-762H8.2	10	0.020	1.19	28.823	5.18
214	ENSG00000049246	PER3	6	0.020	1.11	418.852	158.90
215	ENSG00000120549	KIAA1217	6	0.021	1.18	851.284	212.46
216	ENSG00000185885	IFITM1	6	0.021	-1.12	327.628	1069.18
217	ENSG00000270589	RP11-348N5.7	1	0.022	1.15	18.708	1.69
218	ENSG00000259946	RP11-490G2.2	2	0.023	1.18	38.934	5.70
219	ENSG00000267365	KCNJ2-AS1	1	0.023	-1.17	44.686	201.41
220	ENSG00000260261	RP11-480A16.1	2	0.024	1.12	67.151	18.30
221	ENSG00000027869	SH2D2A	6	0.024	-1.15	44.390	309.26
222	ENSG00000179406	LINC00174	2	0.024	1.03	95.752	33.81
223	ENSG00000258559	AC005519.4	8	0.025	1.16	24.934	5.23
224	ENSG00000143603	KCNN3	6	0.025	-1.16	10.157	49.96
225	ENSG00000112981	NME5	6	0.025	1.12	59.564	18.60
226	ENSG00000164616	FBXL21	11	0.025	1.15	599.239	76.06
227	ENSG00000279265	AC000123.3	9	0.025	1.16	22.785	3.77
228	ENSG00000236841	AC007750.5	1	0.026	1.08	146.043	43.85
229	ENSG00000240053	LY6G5B	6	0.027	1.04	120.998	38.15
230	ENSG00000162595	DIRAS3	6	0.027	1.14	1920.496	261.85
231	ENSG00000117595	IRF6	6	0.027	1.13	216.690	32.25
232	ENSG00000135119	RNFT2	6	0.027	-1.01	61.986	150.39
233	ENSG00000118946	PCDH17	6	0.028	-1.14	95.414	348.18
234	ENSG00000186409	CCDC30	6	0.028	1.11	52.685	15.62
235	ENSG00000261997	RP11-212I21.4	2	0.028	1.15	45.333	6.27
236	ENSG00000227741	RP11-536C5.7	1	0.029	1.03	58.128	19.87
237	ENSG00000158220	ESYT3	6	0.029	1.14	87.611	14.63
238	ENSG00000137491	SLCO2B1	6	0.029	1.13	72.508	8.14
239	ENSG00000206384	COL6A6	6	0.030	1.14	271.928	82.14
240	ENSG00000116106	EPHA4	6	0.030	1.08	128.463	10.48
241	ENSG00000151617	EDNRA	6	0.031	-1.08	637.587	1941.22
242	ENSG00000240288	GHRLOS	1	0.031	1.11	33.928	9.74
243	ENSG00000279500	RP11-21K12.2	9	0.031	1.10	74.997	18.28
244	ENSG00000225920	RIMKLBP2	4	0.032	1.12	23.907	3.50
245	ENSG00000133135	RNF128	6	0.033	1.12	576.556	175.78
246	ENSG00000239653	PSMD6-AS2	1	0.033	1.11	37.264	8.19
247	ENSG00000280067	CTD-2600H12.2	9	0.033	1.12	38.176	8.33
248	ENSG00000102468	HTR2A	6	0.034	1.02	69.577	2.88
249	ENSG00000272808	RP11-66B24.7	5	0.034	1.07	19.649	1.56
250	ENSG00000112149	CD83	6	0.034	1.06	94.216	33.75
251	ENSG00000099984	GSTT2	3	0.036	1.04	30.574	1.69
252	ENSG00000113645	WWC1	6	0.037	1.08	241.574	90.45

Continued on next page

Continued from previous page

	Gene ID	Gene name	Biotype	p_{adj}	M	ACM_{count}	$CTRL_{count}$
253	ENSG00000278991	RP11-797A18.5	9	0.037	1.10	34.900	7.18
254	ENSG00000180229	HERC2P3	12	0.038	1.06	294.427	28.83
255	ENSG00000019582	CD74	6	0.040	1.09	42.868	12.42
256	ENSG00000246859	STARD4-AS1	1	0.041	1.08	405.241	72.36
257	ENSG00000138623	SEMA7A	6	0.041	-1.05	2644.231	9133.89
258	ENSG00000214548	MEG3	2	0.041	1.09	12716.711	2405.79
259	ENSG00000127472	PLA2G5	6	0.042	1.07	114.248	18.47
260	ENSG00000133083	DCLK1	6	0.042	1.06	759.094	311.02
261	ENSG00000230417	LINC00856	2	0.042	1.01	53.084	4.09
262	ENSG00000119630	PGF	6	0.043	-1.05	562.812	1714.26
263	ENSG00000272578	AP000347.2	12	0.045	1.00	56.334	17.58
264	ENSG00000224950	RP5-1086K13.1	2	0.046	1.07	45.872	11.54
265	ENSG00000224259	LINC01133	2	0.046	-1.07	120.786	464.59
266	ENSG00000203872	C6orf163	6	0.046	1.06	36.536	7.95
267	ENSG00000184258	CDR1	6	0.046	1.07	65.681	11.93
268	ENSG00000157152	SYN2	6	0.047	1.03	38.463	4.54
269	ENSG00000233025	CRYZP1	4	0.048	1.06	18.598	2.93
270	ENSG00000135678	CPM	6	0.048	1.05	379.738	108.45
271	ENSG00000250846	EPHA5-AS1	2	0.048	1.05	69.268	20.56
272	ENSG00000255282	WTAPP1	10	0.049	1.06	27.900	7.90

Supplementary Table S10: Genes significantly differentially expressed between ACM and control samples. Columns p_{adj} and M contain (adjusted) p-values for significance and $\log_2$ fold-change values for their extent of differential expression between ACM and control samples. Column *Biotype* lists the biotype of the gene: 1: antisense, 2: lincRNA, 3: polymorphic_pseudogene, 4: processed_pseudogene, 5: processed_transcript, 6: protein_coding, 7: sense_intronic, 8: sense_overlapping, 9: TEC, 10: transcribed_processed_pseudogene, 11: transcribed_unprocessed_pseudogene, 12: unprocessed_pseudogene. Columns ACM_{count} and $CTRL_{count}$ provide the (normalized) average read-counts for the genes across all ACM and control samples, respectively. Genes are ordered by significance.

gene_name	p_{seq}	M_{seq}	p_{pcr}	M_{pcr}
PCDH1	0.00	-3.69	0.01	-3.49
SAXO2	0.00	2.72	0.00	3.81
SEMA3D	0.00	1.89	0.04	2.51
NEDD9	0.00	-1.57	0.01	-2.31

Supplementary Table S11: Differential expression estimates for the indicated genes from real time RT-qPCR experiments (suffix *pcr*) and RNA-seq experiments (suffix *seq*). Listed are p-values (raw p for RT-qPCR and adjusted p-values for RNA-seq) and M-values for the comparison ACM *vs* CTRL. SAXO2 was not detectable by RT-qPCR in control samples and an artificial C_q value of 40 was used thus to enable estimation of its differential expression. RT-qPCR measurements were performed in RNA samples from cells of the individuals also profiled with RNA-seq, but from different passages and experiments.

Gene ontology enrichment analyses.

GO ID	p_{adj}	Count	Size	Term	Gene names
GO:0005887	0.0002	27	765	integral component of plasma membrane	SLCO2B1 JAG1 EDNRA EFNB2 EPHA4 EPHB2 F2RL1 GABRE LRRC32 HTR2A ICAM2 NPR3 PCDH1 PCDH12 PLXNB3 SCN8A HHIP VAMP1 ESYT3 ITGA10 ABCC3 CCRL2 P2RX6 DCLK1 CD83 ABCG1 CD79B
GO:0031224	0.0173	62	3134	intrinsic component of membrane	CDH10 ATP8A1 SLCO2B1 CATSPER2 CPM TMEM229B JAG1 EDNRA EFNB2 ADCY4 EPHA4 EPHB2 F2RL1 ENPP4 SLC7A11 GABBR1 GABRE LRRC32 PCDH17 GPM6B KCNIP3 HTR2A ICAM2 TMC3 KCNJ2 KCNN3 MCAM LRRC37A2 NPR3 NOX4 PCDH1 PCDH12 ABCB1 PLXNB3 MARCH1 PCDHB7 CD248 RXFP1 TMEM35 SCN8A HHIP VAMP1 C5 KCNAB1 RNF128 GALNT14 FRAS1 ESYT3 KREMEN1 SEMA7A RNFT2 ITGA10 IFITM1 KIAA1644 ABCC3 CCRL2 P2RX6 DCLK1 CD83 ABCG1 CD74 CD79B

Supplementary Table S12: Cellular component GO-terms with significant enrichment of genes differentially expressed between ACM and CTRL samples. Column p_{adj} contains the enrichment p-values adjusted for multiple hypothesis testing using the Bonferroni method.

GO ID	p_{adj}	Count	Size	Term	Gene names
GO:0015872	0.0047	5	17	dopamine transport	GABBR1 HTR2A CXCL12 SNCA SNCG
GO:0050432	0.0191	5	22	catecholamine secretion	GABBR1 HTR2A CXCL12 SNCA SNCG

Supplementary Table S13: Biological process GO-terms with significant enrichment of genes differentially expressed between ACM and CTRL samples. Column p_{adj} contains the enrichment p-values adjusted for multiple hypothesis testing using the Bonferroni method.

Gene ID	Gene name	p_{adj}	M	ACM_{count}	$CTRL_{count}$
ENSG00000173801	JUP	0.001	-1.2	3191.0	6778.6
ENSG00000046604	DSG2	0.331	0.5	203.9	26.0
ENSG00000119699	TGFB3	0.644	0.3	228.2	226.2
ENSG00000155657	TTN	0.669	0.3	404.0	277.1
ENSG00000096696	DSP	0.779	-0.2	7263.0	10001.9
ENSG00000160789	LMNA	0.952	0.0	45195.8	51197.9
ENSG00000170876	TMEM43	0.957	-0.0	6062.9	6332.7
ENSG00000057294	PKP2	0.977	-0.0	70.9	63.6

Supplementary Table S14: Genes with ACM associated variants expressed in the analyzed samples. Columns p_{adj} and M list the genes' (adjusted) p-values for significance and $\log_2$ fold-change values for their extent of differential expression between ACM and control samples. Columns ACM_{count} and $CTRL_{count}$ provide the (normalized) average read-counts for the genes across all ACM and control samples. Genes are ordered by significance.

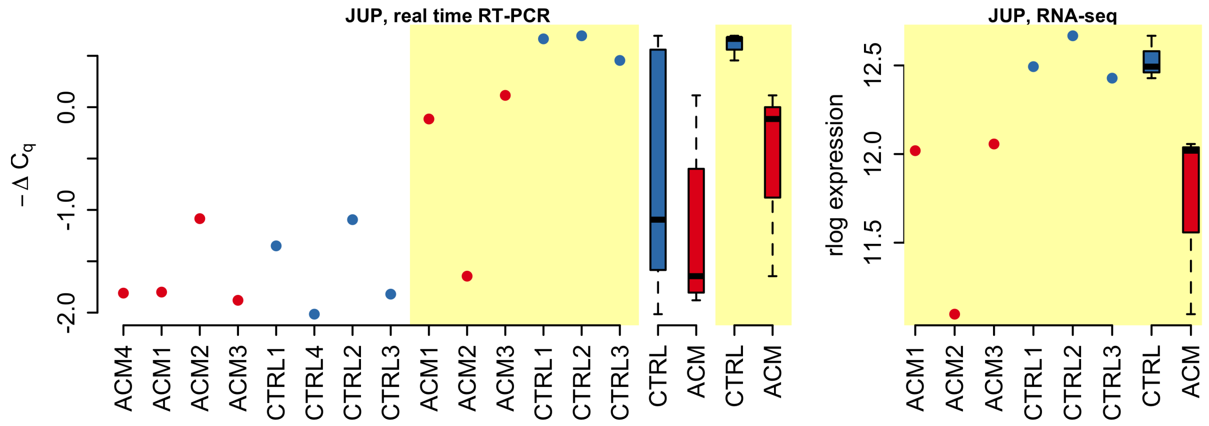

Supplementary Figure S2: Expression estimates for JUP from RT-qPCR experiments (left) and RNA-seq experiments (right). Red and blue coloring indicates ACM and CTRL samples, respectively. Data points plotted on yellow background were measured in the RNA used for RNA-seq based expression profiling, the other data points represent measurements in RNA from other experiments (and other passage numbers).

miRNA	fold change	mature miRNA	p_{adj}	M
miR-574-3p	-5.49	hsa-miR-574-3p	0.513	-0.69
miR-100	-4.34	hsa-miR-100-5p	0.712	0.37
miR-671-3p	-4.01	hsa-miR-671-3p	0.396	-0.94
miR-23b	-2.74	hsa-miR-23b-3p	0.753	-0.57
miR-99a	-2.71	hsa-miR-99a-5p	0.976	0.06
miR-20b	2.71	hsa-miR-20b-5p	0.715	0.53
miR-132	2.73	hsa-miR-132-3p	0.788	-0.34
miR-342-3p	2.74	hsa-miR-342-3p	0.759	-0.32
miR-362-5p	3.8	hsa-miR-362-5p	0.642	-0.74
miR-487b	7.39	hsa-miR-487b-3p	0.386	1.39

Supplementary Table S15: miRNAs reported by Gurha et al. to be differentially expressed in HL-1 mouse cardiac muscle cells after shRNA-mediated knock-down of PKP2 that are expressed also in the present data set. Columns *miRNA* and *fold change*: miRNA ID and fold change as reported in Gurha et al., columns *mature miRNA*, p_{adj} and M: mature miRNA name, adjusted p-value and $\log_2$ fold change value as measured in the present data set.

4 Combined miRNA-mRNA expression analysis

High confidence validated target genes of ACM specific miRNAs miR-29b-3p and miR-520c-3p; for miR-1183 no high confidence validated target genes have been reported.

Gene ID	Gene name	p_{adj}	M	ACM_{count}	$CTRL_{count}$
ENSG00000148848	ADAM12	0.00	0.8	19867.5	9434.4
ENSG00000168256	NKIRAS2	0.01	-0.5	3324.6	4585.4
ENSG00000008517	IL32	0.02	-0.6	4832.6	5866.9

Continued on next page

Continued from previous page

Gene ID	Gene name	p_{adj}	M	ACM_{count}	$CTRL_{count}$
ENSG00000105329	TGFB1	0.05	-0.5	8453.0	13092.4
ENSG00000080573	COL5A3	0.08	-1.0	595.2	1789.0
ENSG00000134871	COL4A2	0.16	-0.7	74183.2	103376.4
ENSG00000197461	PDGFA	0.16	-0.8	739.0	1454.5
ENSG00000116962	NID1	0.17	-0.7	13707.9	23349.6
ENSG00000091409	ITGA6	0.25	0.7	3449.9	1645.8
ENSG00000134013	LOXL2	0.27	-0.4	41910.6	49361.3
ENSG00000113140	SPARC	0.27	-0.4	166823.7	207299.8
ENSG00000187498	COL4A1	0.29	-0.7	89770.9	124311.1
ENSG00000168487	BMP1	0.30	-0.3	3732.1	4638.5
ENSG00000166147	FBN1	0.36	-0.3	92171.8	130676.1
ENSG00000030582	GRN	0.36	-0.3	20698.0	24187.8
ENSG00000125966	MMP24	0.39	0.5	48.2	30.9
ENSG00000113083	LOX	0.46	-0.4	30354.5	43312.1
ENSG00000124151	NCOA3	0.46	-0.2	3968.0	4301.0
ENSG00000092969	TGFB2	0.47	0.5	8387.9	3043.4
ENSG00000112715	VEGFA	0.47	0.3	8370.6	6880.3
ENSG00000168769	TET2	0.51	0.3	1513.3	951.5
ENSG00000100311	PDGFB	0.52	-0.5	102.2	170.5
ENSG00000178585	CTNNBIP1	0.53	-0.3	990.0	1373.4
ENSG00000158050	DUSP2	0.61	0.4	69.0	49.1
ENSG00000105810	CDK6	0.61	-0.3	5537.7	4955.7
ENSG00000143384	MCL1	0.61	-0.2	20639.3	23887.8
ENSG00000170345	FOS	0.63	-0.4	79.0	122.0
ENSG00000179094	PER1	0.64	-0.2	491.1	605.6
ENSG00000119699	TGFB3	0.64	0.3	228.2	226.2
ENSG00000119772	DNMT3A	0.65	0.2	736.7	661.3
ENSG00000090520	DNAJB11	0.66	-0.1	7300.9	7620.3
ENSG00000132780	NASP	0.67	0.3	4221.0	4173.1
ENSG00000118971	CCND2	0.67	0.3	283.7	188.6
ENSG00000070831	CDC42	0.68	-0.1	23392.8	25778.7
ENSG00000168542	COL3A1	0.68	-0.3	79079.5	127110.5
ENSG00000088305	DNMT3B	0.70	0.3	356.7	309.9
ENSG00000149257	SERPINH1	0.70	-0.2	38929.1	45625.6
ENSG00000171862	PTEN	0.72	0.2	4299.9	3382.0
ENSG00000167772	ANGPTL4	0.76	-0.3	2580.5	3518.6
ENSG00000108821	COL1A1	0.77	-0.2	269826.7	358147.0
ENSG00000145675	PIK3R1	0.78	0.2	3410.9	2796.5
ENSG00000171791	BCL2	0.80	-0.2	92.0	136.4
ENSG00000087245	MMP2	0.81	-0.1	144634.1	159633.9
ENSG00000113721	PDGFRB	0.84	-0.2	1221.7	2429.3
ENSG00000068024	HDAC4	0.84	0.1	799.0	843.4
ENSG00000107485	GATA3	0.86	-0.2	259.2	334.0
ENSG00000204262	COL5A2	0.86	-0.2	42935.4	45960.6
ENSG00000138131	LOXL4	0.87	-0.2	616.7	855.8
ENSG00000088808	PPP1R13B	0.88	-0.1	542.9	549.2
ENSG00000185669	SNAI3	0.88	0.2	18.8	16.2
ENSG00000082701	GSK3B	0.91	-0.0	5908.7	5467.6
ENSG00000186318	BACE1	0.91	-0.0	7347.7	8463.4
ENSG00000138336	TET1	0.94	0.1	100.3	84.4
ENSG00000091831	ESR1	0.94	0.1	182.7	176.1
ENSG00000139372	TDG	0.94	0.1	2476.0	1892.1
ENSG00000150093	ITGB1	0.95	0.0	160923.2	145888.0
ENSG00000134853	PDGFRA	0.95	0.1	12639.8	12848.8
ENSG00000123500	COL10A1	0.96	-0.1	13.0	14.6
ENSG00000130816	DNMT1	0.96	-0.0	7854.4	9824.9
ENSG00000149948	HMGA2	0.97	-0.0	11148.9	10672.3
ENSG00000145431	PDGFC	0.97	-0.0	3383.6	3291.4
ENSG00000185591	SP1	0.98	-0.0	4140.9	4350.5
ENSG00000105221	AKT2	0.99	-0.0	6439.4	7136.6
ENSG00000116560	SFPQ	0.99	-0.0	16618.4	18193.2
ENSG00000058085	LAMC2	0.99	0.0	631.1	626.4
ENSG00000102996	MMP15	0.99	-0.0	1479.8	1271.3

Continued on next page

Continued from previous page

Gene ID	Gene name	p_{adj}	M	ACM_{count}	$CTRL_{count}$

Supplementary Table S16: Differential expression data (ACM *vs* CTRL) for high confidence target genes of miRNA miR-29b-3p. Genes are ordered by significance of differential expression.

Gene ID	Gene name	p_{adj}	M	ACM_{count}	$CTRL_{count}$
ENSG00000142192	APP	0.01	-0.3	65234.1	77342.2
ENSG00000114867	EIF4G1	0.30	-0.3	46109.8	59143.9
ENSG00000198793	MTOR	0.58	-0.1	5537.2	5986.3
ENSG00000026508	CD44	0.91	0.1	56279.4	54691.3
ENSG00000096717	SIRT1	0.94	0.1	1296.8	1141.7
ENSG00000204520	MICA	0.99	0.0	3952.3	3825.2

Supplementary Table S17: Differential expression data (ACM *vs* CTRL) for high confidence target genes of miRNA miR-520c-3p. Genes are ordered by significance of differential expression.

miRNA	Gene ID	Gene name	Biotype	M_{RTPCR}	$p_{adj,RNAseq}$	M_{RNAseq}	$CTRL_{count}$	ACM_{count}
miR-29b-3p	ENSG00000203709	C1orf132	lincRNA	3.02	0.097	0.68	62	117
miR-29b-3p	ENSG00000226380	AC058791.1	lincRNA	3.02	0.824	0.21	428	537
miR-1183	ENSG00000105866	SP4	protein_coding	4.01	0.438	0.39	165	249

Supplementary Table S18: Potential host genes for ACM specific miRNAs. Columns M_{RTPCR} and M_{RNAseq} contain the log2 fold change values represeting the differential expression between ACM and CTRL samples of the mature miRNA respectively their predicted host gene. Column $p_{adj,RNAseq}$ contains the adjusted p-values for the significance of the latter and columns $CTRL_{count}$ and $\mathrm{ACM}_{\mathrm{count}}$ the normalized read counts for the host gene from the RNA-seq experiment.

5 Network analysis

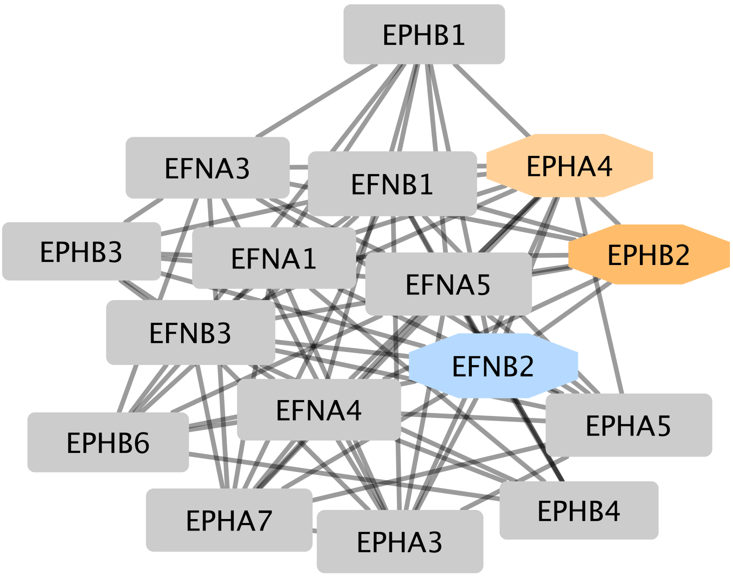

Supplementary Figure S3: Cluster c0703 identified by ClusterONE in the gene network of all genes expressed according to the RNA-seq data set and the protein atlas. Edges between the nodes represent functional relationships, as defined by STRING, or protein interaction as derived from BioPlex and mentha. Yellow and blue coloring indicates significantly up- and down-regulated genes in ACM.
